# Single-Sensor Source Localization Using Electromagnetic Time Reversal and Deep Transfer Learning: Application to Lightning

**DOI:** 10.1038/s41598-019-53934-4

**Published:** 2019-11-22

**Authors:** Amirhossein Mostajabi, Hamidreza Karami, Mohammad Azadifar, Alireza Ghasemi, Marcos Rubinstein, Farhad Rachidi

**Affiliations:** 1 Electromagnetic Compatibility Laboratory, Swiss Federal Institute of Technology (EPFL), Lausanne, Switzerland; 2 Institute for Information and Communication Technologies, University of Applied Sciences of Western Switzerland (HES-SO), Yverdon-les-Bains, Switzerland; 3 ELCA Informatik AG, Zürich, Switzerland

**Keywords:** Electrical and electronic engineering, Computer science

## Abstract

Electromagnetic Time Reversal (EMTR) has been used to locate different types of electromagnetic sources. We propose a novel technique based on the combination of EMTR and Machine Learning (ML) for source localization. We show for the first time that ML techniques can be used in conjunction with EMTR to reduce the required number of sensors to only one for the localization of electromagnetic sources in the presence of scatterers. In the EMTR part, we use 2D-FDTD method to generate 2D profiles of the vertical electric field as RGB images. Next, in the ML part, we take advantage of transfer learning techniques by using the pretrained VGG-19 Convolutional Neural Network (CNN) as the feature extractor tool. To the best of our knowledge, this is the first time that the knowledge of pretrained CNNs is applied to simulation-generated images. We demonstrate the skill of the developed methodology in localizing two kinds of electromagnetic sources, namely RF sources with a bandwidth of 0.1–10 MHz and lightning impulses. For the localization of lightning, based on the experimental recordings in the Säntis region, the new approach enables accurate 2D lightning localization using only one sensor, as opposed to current lightning location systems that need at least two sensors to operate.

## Introduction

Time reversal (TR) has received increasing attention in the field of source localization for applications in medicine, acoustics, electromagnetics, etc. In electromagnetics, for example, several studies have investigated the use of electromagnetic time reversal (EMTR) as a means of locating lightning (e.g.^1–4^). Mora *et al*.^2^ and Lugrin *et al*.^3^ proposed an algorithm to locate lightning discharges that requires at least three field sensors and the accuracy of the lightning localization estimations was investigated under ideal and also lossy propagation conditions. Wang *et al*.^5^ applied EMTR to estimate the direction of arrival of lightning radiation sources. Their proposed method was used to map the progression of the whole lightning discharge.

Although EMTR has been proved to have high accuracy in identifying the electromagnetic source locations, for it to yield a good performance, a sufficient number of sensors are required. For example, while the EMTR-based lightning localization approach developed by Mora *et al*.^2^ has yielded excellent accuracy when 3 sensors are used, it does not successfully locate lightning if the number of sensors is lower than 3.

Machine learning algorithms such as neural networks could give computers the ability to learn a skill (such as the prediction of the geographic coordinates of a passive or active object) from sets of archived data and to apply the skill on new unseen data. Recently, machine learning has been also shown to be a useful method for source localization. For example, Huang *et al*. applied deep neural networks to acoustic source localization in shallow water environments^6^. Vera-Diaz *et al*. used deep learning to directly estimate the three-dimensional position of a single acoustic source using raw data from microphone arrays^7^.

Here, we show as a proof of concept that EMTR and ML can be effectively combined to accurately localize electromagnetic sources using only one electric field sensor. In the EMTR localization technique, usually three steps are defined: (i) several transducers placed in different locations are used to record the electric or magnetic field from the source, (ii) the time domain signals recorded by each of the sensors are then time-reversed and synchronously retransmitted back into the medium, and (iii) the results from the back-propagation step are analyzed to determine the source location^8^. The main idea of this paper is to substitute the conventional methods that are used to make a localization decision based on the back-propagation results (e.g., energy and cross correlation) with machine learning techniques. To do that, the 2D electric field profiles from the output of the back-propagation phase in the EMTR technique are first converted to RGB images and then transmitted to the pretrained VGG-19 Convolutional Neural Network (CNN) in order to extract an efficient feature vector representing these images^9^. Two regressors are then trained, each to estimate the x and y coordinates of the source localization based on the aforementioned extracted features.

Moreover, in some of the studies related to EMTR^2–5,10,11^, the 1/R dependence of the radiated wave in the back-propagation step is removed in order to deal with singularities at the source locations. Back-propagation of the fields by keeping their amplitude constant ensures that the total field will be maximum at the location of the original source and that the fields from the individual sensors will not vanish at very large distances. The maximum constructive interference will necessarily occur at the source point because it is only there that the relative phase delays will vanish if three or more sensors are used. However, forcing the amplitude of the propagating wave to be constant by eliminating the 1/R dependence has two main disadvantages: (i) the condition cannot be applied to numerical methods that are commonly used in many electromagnetic simulators, and (ii) the condition is not applicable in the presence of one or more scatterers in the computational domain. To avoid these disadvantages, some papers have abandoned the condition of constant amplitude and have used instead the entropy technique to find the optimum time slice and then obtain the source location^1,12^.

In this paper, we combine EMTR and Machine Learning to locate radiation sources. We call the combined methodology EMTR/ML. In the EMTR part of the methodology, we use the Two-Dimensional Finite Difference Time Domain (2D-FDTD) method to calculate the direct and back-propagated waves. The simulation results obtained with the full-wave 2D-FDTD method intrinsically include the propagation losses in the forward and backward time and they improve the accuracy of the source geolocation. Moreover, scattering objects such as mountains can be readily included in the FDTD simulation space. While a 3D model is able to better represent the complexities inside the medium, such as the terrain topography, the actual shape of the scatterers, and the ground conductivity, it is computationally more expensive and time consuming compared to 2D simulations. Hence, in this paper, simple 2D modeling of the scattering objects is used to perform the analyses. We have shown that even using such a simplified model, the proposed method can provide reasonable localization accuracy.

In order to validate the methodology, two case studies are defined. In the first case study, the goal is to localize a Gaussian RF source with 0.1–10 MHz bandwidth based only on numerical simulation results. In the second case study, the methodology is used to find the location of lightning strikes using both numerical and experimental data. In both cases, we will show that only one electric field sensor is needed to reach a reasonable localization accuracy. The presence of mountains or other scatterers is required for the method proposed in this paper to locate the source, since it uses measured fields only at one location.

## Methods and Results

### Case study 1

**Figure 1:**
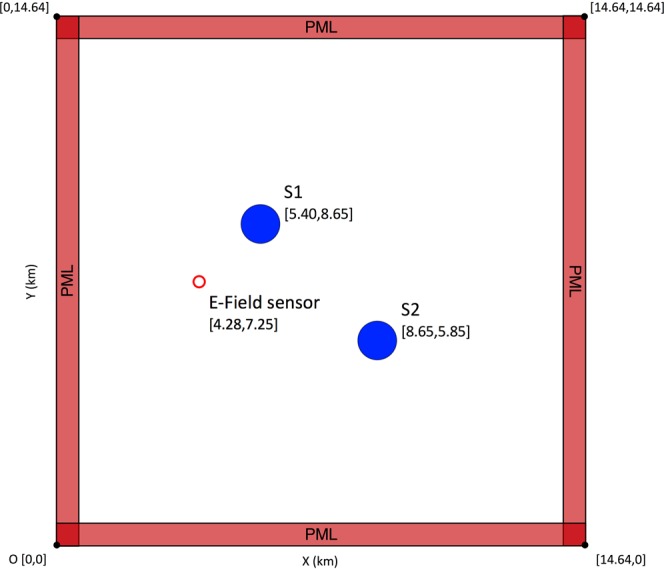
Geometry of the problem in the first case study. The blue/filled circles and the red/unfilled circle show the scatterers and the location of the electric field sensor, respectively. The solution space spans 13.44 × 13.44 km^2^.

The proposed methodology is first applied to localize a Gaussian RF source with a 10 MHz bandwidth using only one electric field sensor. First, the EMTR and ML methods are applied separately to the problem. The results are then compared with that of the proposed combined approach to evaluate the obtained improvement. We used a simple yet realistic geometry as the detection region. It consists of one electric field sensor and two cylindrical scatterers with a radius of 500 m which are put inside the medium as shown in the schematic diagram in Fig. 1.

#### Numerical simulation using EMTR

**Description of the procedure**: Consider the 2D geometry shown in Fig. 1. In the EMTR approach, first, a random position for the source was selected. The source was a z-axis dipole with a Gaussian pulse current source of 10 MHz bandwidth and 1 A/m current density amplitude. Then, the incident electric field at the location of the sensor was obtained by means of the 2D-FDTD method. Perfectly Matched Layers (PML) with a depth of 10 mesh cells were deployed as boundary conditions at the perimeter of the simulation domain. Equally spaced cells with the length of 60 m were used to mesh the solution space.

**Figure 2:**
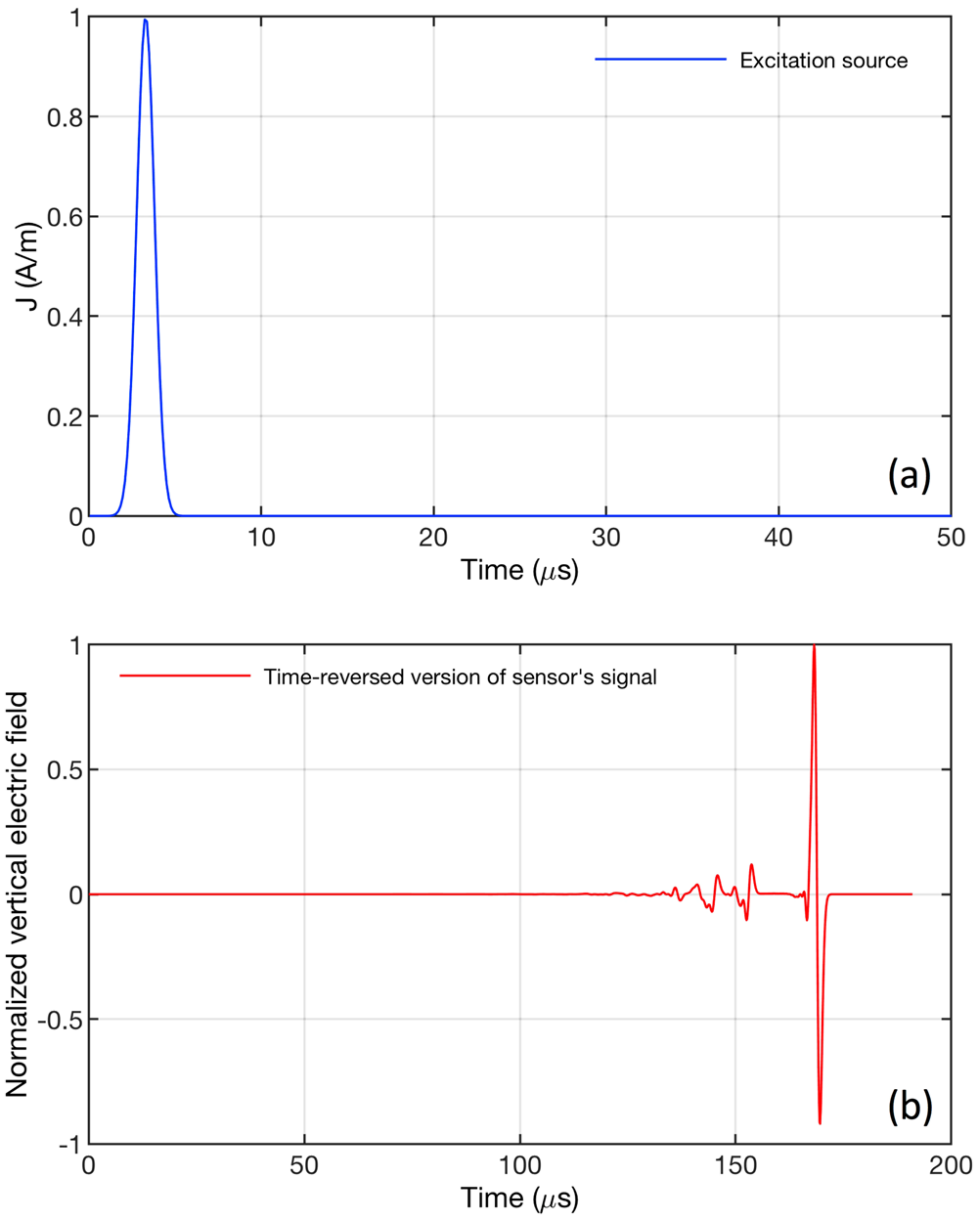
Example of the simulation results for the source and the field at a sensor. (**a)** The linear current density of the excitation source (Gaussian RF with 10 MHz bandwidth) and (**b)** the time-reversed version of the signal measured by the electric field sensor normalized to its maximum value.

The electric field at the sensor’s location was normalized to its maximum value. The end of the sensor signals was set to the time at which the derivative with respect to time of the normalized signal’s energy becomes smaller than 10^−9^ 1/s. The recorded signal was then time-reversed and back injected into the medium. Figure 2 shows an example of the current density for the excitation source as well as the corresponding time-reversed version of the electric field calculated at the sensor and normalized to its maximum value. The FDTD method was used again in the back-propagation to calculate the distribution of the electric field inside the medium. Using the maximum amplitude criterion, the EMTR method assumes the source position to be the point with the maximum amplitude of the vertical electric field over the whole simulation time window.

**Figure 3:**
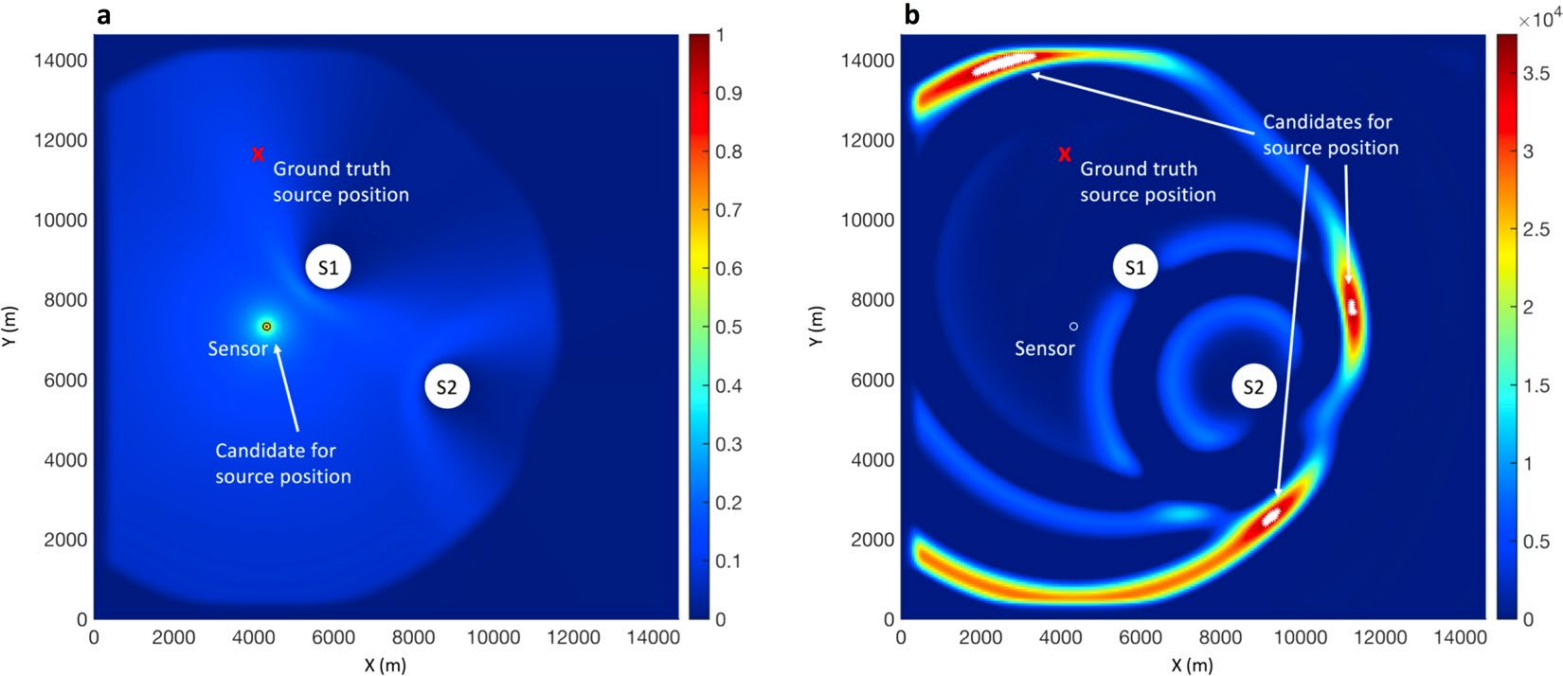
**a**) Maximum amplitude of vertical electric field at all time steps normalized to its maximum value and (**b**) 2D profile of the vertical electric field at the last time step. The red cross is the ground truth of the source position, S1 and S2 are the scattering objects, and the white circle is the electric field sensor. The nominated point/points by EMTR are also annotated on each panel.

**Results**: The normalized maximum amplitude of the electric field at all time steps is given in Fig. 3a. As shown in that figure, the maximum amplitude of the electric field occurs at the location of the sensor, which is not the correct source position. This failure to locate the original source was also reported in Karami *et al*.^1^ and it was attributed to the consideration of the 1/R propagation factor in the EMTR calculation.

Moreover, even when the 1/R propagation factor is ignored, the results from several previous studies (e.g. Mora *et al*.^2^ and Lugrin *et al*.^3^) showed that by using less than three sensors, the EMTR algorithm leads to multiple positions in the time-reversed wavefront with the same value of the electric field. Instead of getting one unique point, a line of multiple candidates of the source point is observed.

Instead of looking at the results over all of the time steps, some previous studies used the EMTR back-propagation stage results at a specific time slice during the simulation time window to find the source. For example, Karami *et al*.^1^ used the entropy criterion to find the optimal time slice among the back-propagation time steps to locate the lightning strike point. The point with maximum electric field in the selected time slice would correspond to the source position. While this method was successful in localizing sources using 2 and more sensors, it was not applicable in our case study when only one sensor is involved as it led to an ambiguity in the response. To show this ambiguity, let us consider the simulation presented in Fig. 3a but instead of plotting the maximum electric field over the whole simulation time, let us examine the 2D profile of the electric field at the last time step (i.e., the moment when the back-propagation ends). The resulting plot is shown in Fig. 3b. In this figure, there are three regions of high electric field inside the 2D profile and each includes several candidates for the source position based on the EMTR approach. Therefore, instead of getting one unique point, a number of multiple candidates of the source point is identified. A similar behavior can be seen if the results from other time slices are considered. This ambiguity was also reported in Mora *et al*.^2^ when less than 3 sensors were used to locate the lightning strike point using EMTR. Furthermore, none of the nominated points corresponds to the actual source position.

#### Numerical simulation using machine learning

**Building the database**: Machine learning is an effective approach for approximating a function from a finite set of its input-output pairs, when such approximation cannot be done through parametric estimation or the function has no closed form. In this section, we aim to train a machine learning model to estimate the geolocation of an electromagnetic source, given the data on the incident electric field at a single remote electric field sensor. To do that, we defined more than 1600 random positions for the source within the geometry presented in Fig. 1. In order to form the required database for training and testing procedures, for each of these source positions we carried out the following steps:

(1) We placed the Gaussian RF source plotted in Fig. 2a at the selected position and we performed the 2D-FDTD simulation described in Section I.A.1 but only in the direct phase to calculate the vertical electric field in the time domain at the location of the sensor.

(2) Next, the values were normalized to the maximum recorded value. This normalization was done to make sure the results would not be sensitive to the amplitude of the source signal. Similar to Section I.A.1, we continued to record the sensor signal until the time derivative of the normalized signal’s energy became less than a predefined threshold.

Once all random positions were considered, a preprocessing step was required to make all recorded sensor signals be of the same length. We did so by padding shorter signals with zeros up to a size of N_max_ (i.e., the maximum length among the recorded signals).

We formed a tabular database out of the processed recorded incident electric field values. In each row, the corresponding samples of the normalized electric field measured at the place of the sensor were used as the predictors and the x and y coordinates of the source were used as the response.

**Machine learning modeling**: Once the database was formed, a machine learning algorithm was employed to identify regularities between predictors and responses using a portion of the data which is known as the training set. The model could then use the explored correlations to predict the response for the unseen cases (testing set). A model search process was conducted to choose the most appropriate machine learning regression model. The candidate models were (i) several regression types including regression trees, support vector machines, and gaussian process regression models, (ii) different ensemble methods such as bagging and boosting, and (iii) the neural networks. The results showed that for the prediction of both, the x and y coordinates, the best performance in the sense of the Mean Squared Error (MSE) was achieved using the XGBoost algorithm. Extreme Gradient Boosting (XGBoost) is a variant of the gradient boosting machine which uses a more regularized model formalization to control overfitting^13^. More information on the XGBoost model description and generation can be found in^13^.

**Model training and testing**: In this study, the predictive ML model was evaluated using a 5-fold cross-validation described as follows. First, the dataset was shuffled and split into five different groups. As a result, each observation in the dataset was assigned to an individual group and remained there for the duration of the training and testing processes. Each unique group was held out from the dataset as the test set and the remaining four groups were used as the training set. The model was then fitted on the training set and evaluated on the test set. The process was repeated until each individual group had been taken once as the test set. The model outputs were combined over the rounds. This splitting method helps to eliminate the leakage of correlated samples from the training set into the testing set. Moreover, it avoids overfitting to a specific testing set since each one of the observations is considered as part of the testing set in one of the five rounds. Once the five rounds of training and testing were done, the model’s prediction skill was evaluated by comparing the outputs with the target values for the x and y coordinates.

**Figure 4:**
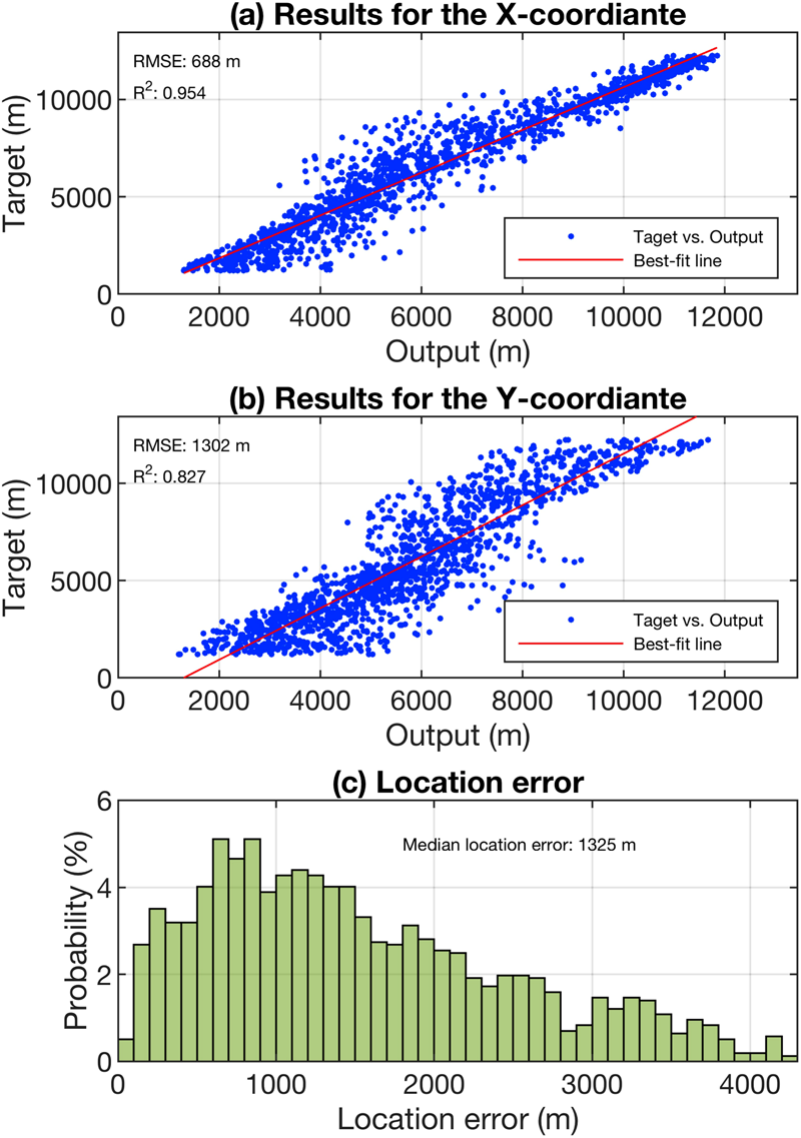
Model performance results for the case of numerical simulations using machine learning. (**a**) Estimation of the x coordinate and (**b**) the y coordinate of the randomly selected source locations. (**c**) Histogram of the location error.

**Model evaluation results**: The model estimations and actual responses were compared to evaluate the model’s prediction accuracy. In Fig. 4a,b, the evaluation results are presented by means of scatter plots of target versus predicted values for the x and y coordinates. A histogram of the Euclidean distance between the target and the ground truth for each of the considered source positions is given in Fig. 4c. In order to visualize the error, 2.5% of the total dataset that had the largest localization errors compared to the remaining locations were considered outliers and excluded from the evaluation results (the same is done in the rest of the paper). The best-fit lines are plotted using the robust least-squares regression method with bisquare weights^14,15^. The method iteratively reweighted the least-squares algorithm to minimize a weighted sum of squares. At each iteration, the robust weights are computed and given to each data point based on how far it is from the fitted line. In this way, the data are fitted using the usual least-squares approach while the effects of outliers are minimized at the same time^16^. The complete procedure followed by the bisquare method can be found in MathWorks User’s Guide^16^. Looking at the results in Fig. 4, one can see that the model shows poor estimation skill on the data with a median localization error greater than 1300 m. The model’s estimation skill is worse on the y coordinate compared to the x coordinate with the coefficient of determination (R^2^) decreasing from 0.954 to 0.827 and the Root Mean Squared Error (RMSE) increasing from 688 m to 1302 m.

#### Numerical simulation using the combination of EMTR and machine learning

The results in Figs 3 and 4 show that applying each of the described EMTR and ML methods separately to the localization problem can lead to inaccurate results when only one sensor is used. However, by looking more closely into the results, one can see that it is possible to improve the performance of the methods. For example, the ML approach could be improved by feeding more relevant input features rather than just the transient electric field waveform. The back-propagation stage in EMTR is helpful in this regard as it transforms the 1D sensor’s signal data into a sequence of 2D arrays (i.e., the profile of the electric field over the detection region and over the back-propagation time window), which includes details regarding the medium such as asymmetries in the location domain (caused by mountainous terrain, scatterers, etc.).

Therefore, teaming up these two methods can make up for the weaknesses of each one and hence increase the performance results. What follows explains the combinational approach proposed in this study.

**Building the database**: The procedure starts with the building of a database. To that end, the following steps were carried out using the same geometry used in sections I.A and I.B. First, we generated a pool of random source positions (N > 1200) and we followed the steps described in Section I.B.1 to form a tabular database out of the sensor signals. Next, instead of simply using these signals as the input for a ML model, we back-propagated the time reversed signal from the location of the sensor using the 2D-FDTD method. At the end of the back-propagation stage, we looked at the 2D array of the vertical electric field associated with the last time step over the detection region. Second, we normalized the vertical electric field values by the maximum value in the 2D array, generated a 2D surface plot out of the data and saved it as an RGB image using the jet colormap array. We then continued with the next source position in the list to generate the next sample image. These images were treated as the sources of valuable visual features in the next steps in the algorithm. A sample of the produced images is shown in Fig. 1. One should note here that in this figure, the positions of the scatterers, the excitation source, and the sensor are annotated only for the sake of presentation and were not included in the version given to the CNN.

**Feature extraction**: Given our dataset of the images with a size of 224 × 224 pixels each, a traditional feedforward neural network would require 50176 input weights which would require a very long training time and a large amount of training data. Furthermore, a flattening of the image matrix of pixels to a vector loses the spatial structure in the image, which contains valuable information about scatterer positions, field distributions, etc. To bridge this gap, instead of using the pixel values as the features, Convolutional Neural Networks (CNNs)^17,18^ can be used to automatically learn semantically meaningful features from images. CNNs contain multiple hidden layers, each learning features in increasingly higher levels of semantic granularity from an image. This is achieved by applying various filters to the training image and transmitting the convolved image as the input to the next layer. More information about different types of layers in CNNs and their functionalities can be found in^19^.

Although CNNs are proven to be powerful tools in working with data coming in the form of multi-dimensional arrays (e.g.^20–29^), there are some limitations if one wants to train the weights in these networks from scratch: (i) a huge number of examples are needed for the network to understand the variation of features, (ii) the training process requires intensive computational resources and it is usually time consuming, and (iii) configuring the network architecture from scratch could be overwhelming due to the existence of many combinations of network layers. Looking at the architecture of CNNs reveals that they learn in a hierarchical manner^30^. This means that features detected by the first layers are more generic and can be reused in different problem domains, while features computed by the last layers are specific and depend on the chosen dataset and task. This has inspired researchers to take advantage of the available powerful pretrained CNNS, already trained to extract effective features from a huge number of training images, in order to solve tasks rather than the original target for which these networks are trained. In this regard, the internal stages of such pretrained CNNs can be used as a feature extractor tool to detect certain low-level features (that could be shared between the images), such as edges, shapes, corners, and brightness from new collections of images. These extracted features would then be transmitted to a second network to learn the target task. This process is an example of “transfer learning” (e.g.^31–33^), in which the representational power of pretrained CNNs can be used to extract some general features from images and hence accelerate the training procedure. This approach is especially of substantial help when the number of available training images is limited and/or the computational resources are not sufficient to train deep learning models from scratch.

In this study, we took advantage of transfer learning to extract features from the generated images using pretrained convolutional networks. As the feature extractor in this study, we imported the Oxford VGG-19 convolutional neural network^9^ with the pretrained weights on the ImageNet database^34,35^ as implemented in the Keras library. The network is 19 layers deep with an image input size of 224 × 224. This network has been trained on more than a million images with the original goal of classifying images into 1000 object categories. In order to repurpose the network for our needs, we cut the top layer of the VGG-19 model (i.e., the classifier) and used the rest of the layers as the feature extraction mechanism. By testing each of the images with the repurposed pretrained model, we got a 4096-dimensional feature vector for each image. These vectors were then concatenated to form the dataset for the regression models which further learn to estimate the x and y coordinates of the source positions, as explained in the next section.

**Regressors on Top of the Pretrained CNN**: Once the features were extracted from the images by means of the pretrained VGG-19 network, custom regression layers were needed to do the source localization based on these extracted features. In this regard, we replaced the top layer of VGG-19 with several trainable layers to estimate the x and y coordinates of the source location. Parameter tuning was conducted through cross-validation, where the validation set was separated from both the test and the training sets. In order to make the optimization tractable, we approached the tuning in a greedy manner. We started with the number of layers and we concluded that the best results are achieved with a single hidden layer. We then optimized the number of nodes in the hidden layer, reaching an optimum for 20 nodes. The next step was fixing the gradient descent algorithm, choosing from the set of possible implementations provided by Keras^36^. Adadelta^36^ proved the best here, surpassing others with a significant margin. The last step was finding the appropriate activation function; the rectified linear unit (ReLU) function achieved the best result and was chosen for the model.

**Figure 5:**
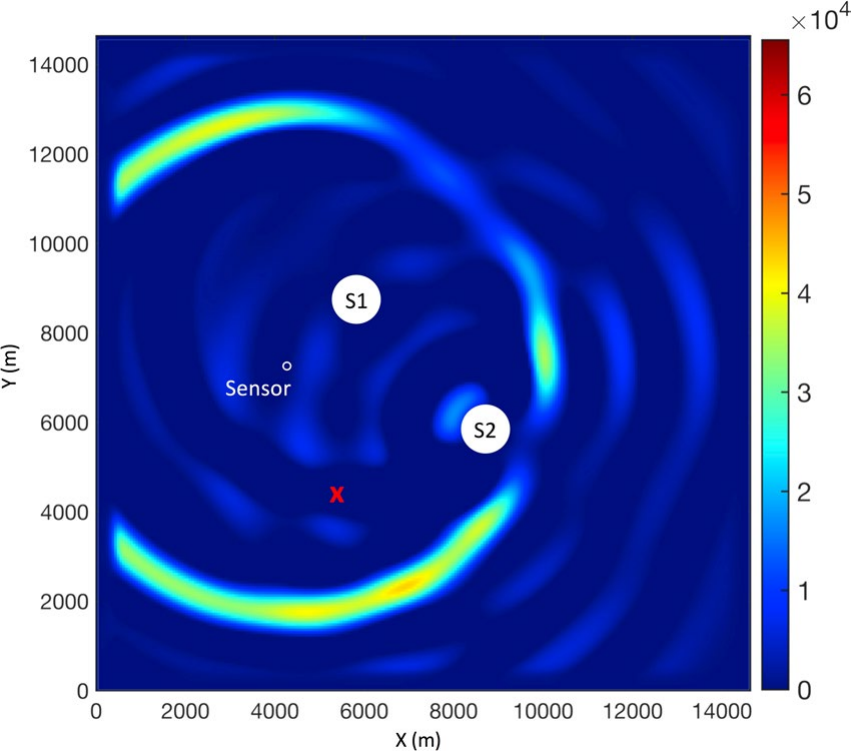
A sample of the generated images out of the 2D surface vertical electric field values in the detection region. The corresponding source position is shown as a red cross on the image. The images are produced using the jet colormap array with 2^16^ elements.

In order to better understand the internal representation that the model has from an input image, the feature maps from the five main blocks of the VGG-19 model are presented in Figs S1–S5 (Supplementary Material). The input image is the one presented in Fig. 5 and the feature maps correspond to the first 64 outputs of the last layer in each block (i.e., layers 2, 5, 10, 15, and 20). As seen in the heat maps of the filter responses in layers 2 through 20 plotted in Figs S1–S5, the filters in the VGG19 layers indeed look for rings of certain sizes and in various locations in the case of EMTR images. From a physical point of view, each ring represents the source response with a certain intensity. However, from a purely data-driven, statistical point of view, namely the way the neural architecture operates, some of the rings can be “computed” from the others and, hence, they are ignored by the network, thereby making the estimation with a finite number of nodes possible.

Continuing this process in consecutive layers, the response maps in the consecutive layers of the network are gradually consolidated into a single value, using a pooling layer in the later stages of the feature engineering network. The computed feature vector is then, very roughly, an indicator of whether a ring with a certain size, location, and intensity has been present in the image or not and, if that is indeed the case, how bright it has been. A super-linear combination of such responses, as learned by the single-layer neural network, estimates the location of the source.

**Training, validation, and testing procedure**: With these new top layers, the predictive neural network was trained, validated and tested using a 5-fold cross validation, as described previously. Fifteen percent of the training samples (i.e., 12% of all samples) were separated and used as the validation set. The model was then fitted on the training set and the performance was evaluated on the validation set at each epoch. The model with the minimum loss on the validation set was used to retain the estimation results on the test set. The model outputs were combined over the rounds. The use of a validation set enables us to make an unbiased evaluation of the model fit while, at the same time, fine-tuning the model hyperparameters. It also helps to keep the test set as an independent subset of data which is only used once the model is completely trained using the training and validation sets. Moreover, the use of cross-validation as the evaluation method avoids overfitting to a specific testing set since each one of the observations is considered as part of the testing set in one of the five rounds. Once the five rounds of training and testing were completed, the model’s prediction accuracy was evaluated by comparing the outputs with the target values for the *x* and *y* coordinates.

**Figure 6:**
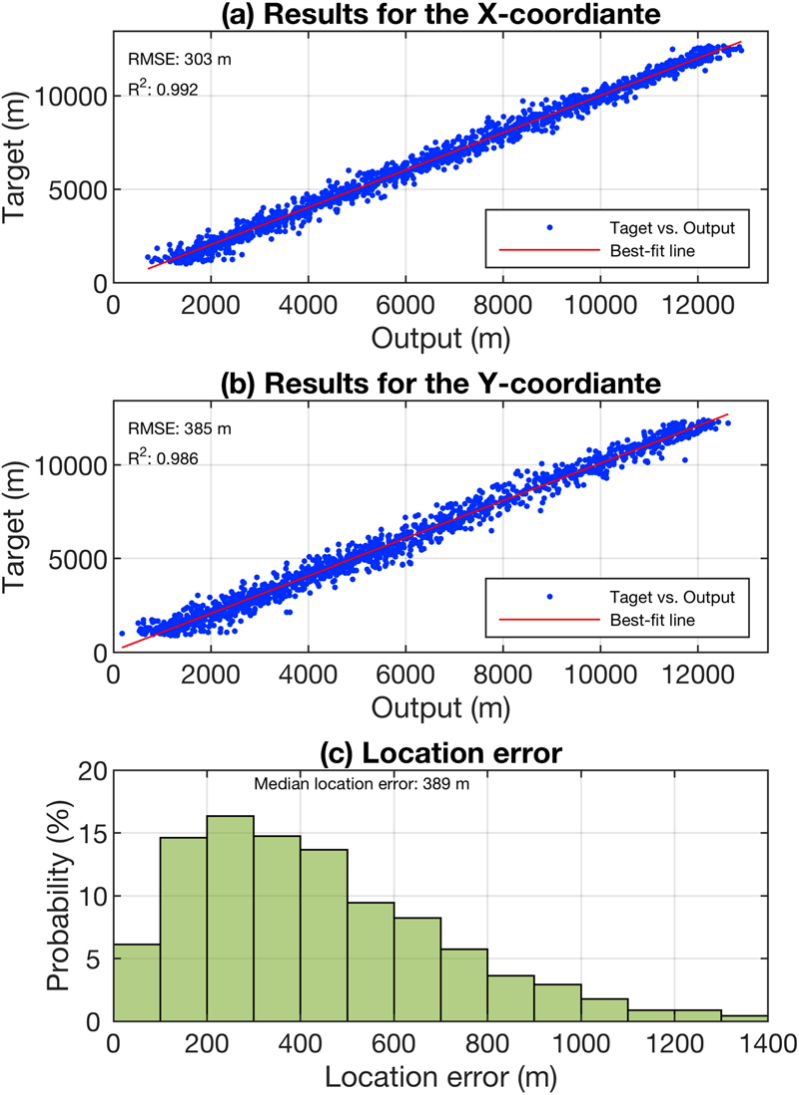
Model performance results for the case of numerical simulations using combinational EMTR/ML approach. The detection region is shown in Fig. 1 including two scattering objects. (**a**) Estimation of the x coordinate and (**b**) the y coordinate of the randomly selected source locations. (**c**) Histogram of the location error.

**Model evaluation**: Figure 6a,b present scatter plots of the target versus output values in which the blue dots correspond to the observations in the database. The best-fit lines are calculated using the least-squares regression method. The very high values of the coefficient of determination (R^2^) indicate that a high proportion of the variance in the data is explained by the fitting line. The results in Fig. 6a,b show that the Root Mean Squared Errors (RMSE) are 303 m and 385 m for the x and y coordinates, respectively. These low error values reveal that despite having used only single-site electric field data, the model was able to accurately predict the location of the nearby RF source along the x and y axes. Finally, the relative probability of the location errors between the ground truth 2D source position and the estimated one by the model for each of the considered source positions is given in the histogram of Fig. 6c. The height of the bar in each bin is the relative number of observations that fall into that bin. According to the results, in more than 75% of the cases, the model was able to predict the source with less than 600-m location error and the median location error was 389 m.

**Figure 7:**
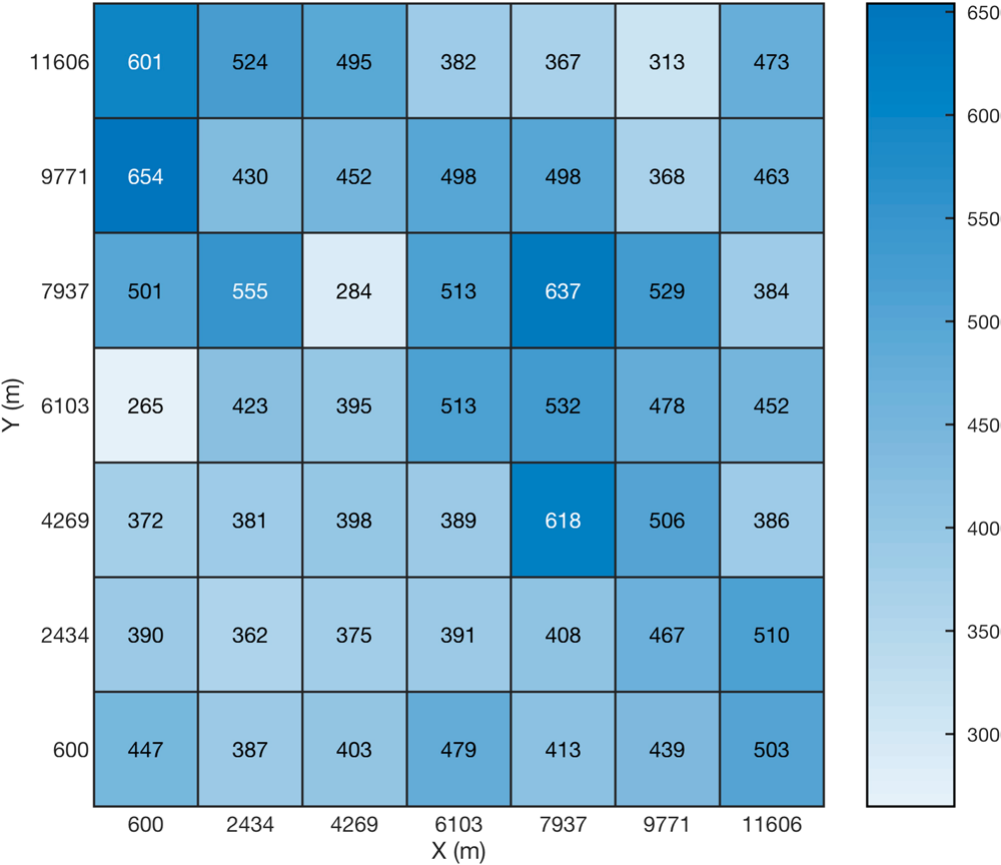
Average location error presented as a heatmap chart inside the detection region for the case of numerical simulations using the EMTR/ML combinational approach. The size of the scatterers, their number and locations, and the frequency of the excitation source remained fixed for simulations at each of the source locations. The colormap represents the location errors in meters. The (0,0) point corresponds to the coordinate center (O) shown in Fig. 1. The x and y labels for each of the cells are the coordinates of the bottom-left corner of the grid cell.

In order to visualize the location error as a function of the source position inside the detection region, a heatmap of location errors is shown in Fig. 7. The figure shows, for each grid cell, the average of the location errors estimated by the model for the samples that fell into that cell. The colormap scale indicates the location error in meters. The 0 values around the perimeter correspond to the PML marginal layer (see Fig. 1).

**Sensitivity to the source and medium parameters**: So far, the size of the scatterers, their number and locations, and the frequency of the source remained fixed. In practical cases, knowledge of these parameters may be imperfect or incomplete. We therefore investigated the sensitivity of the model to variations in these parameters by redoing the analysis described in Section I.C with the following changes to the detection region shown in Fig. 1 before building the database: (i) either a third scatterer was added randomly to some of the cases with its center at [8.05, 8.05] (making the overall number of scatterers be 3), or the second scatterer, S2 in Fig. 1, was removed randomly from some of the cases (i.e., only S1 exists in the medium), (ii) the radius of each of the scatterers was selected independently between 100–500 m, (iii) the frequency of the Gaussian RF excitation source was changed randomly to values between 100 kHz and 10 MHz, and (iv) the number of source locations was increased to more than 6700. The rest of the analysis, including the model generation, training, validation and testing methods were the same as the ones explained in sections I.C.2 and I.C.3.

**Figure 8:**
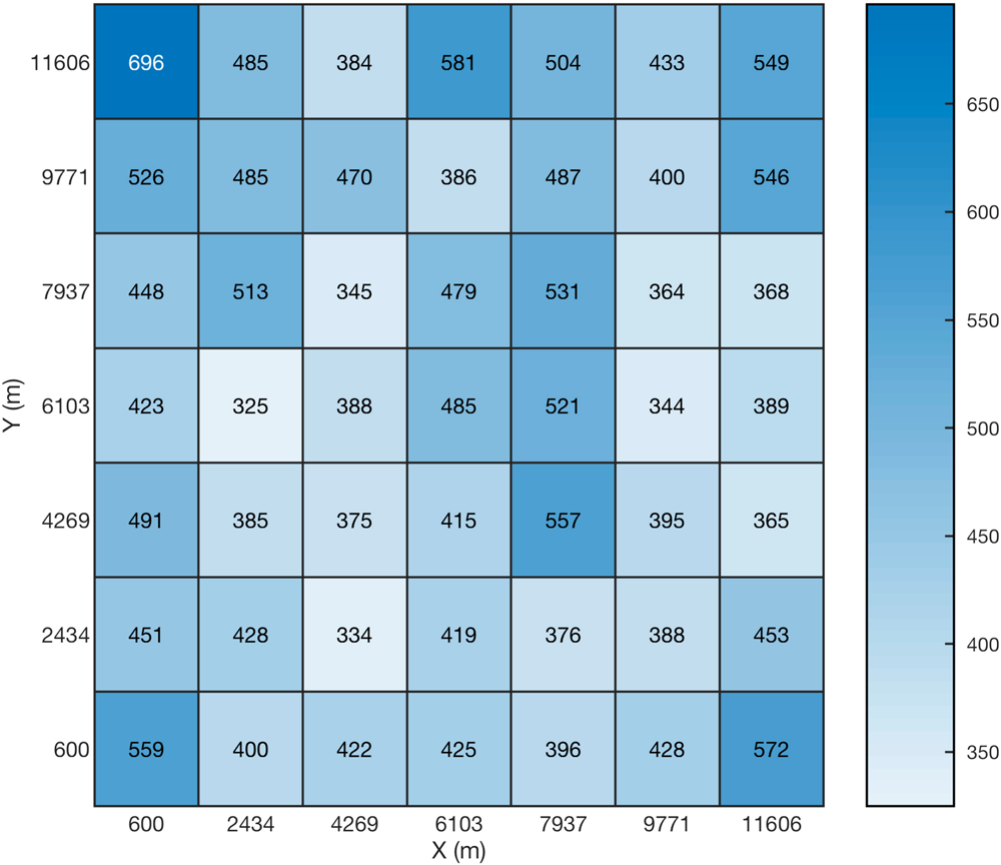
Average location error presented as a heatmap chart inside the detection region for the case of numerical simulations using the combinational EMTR/ML approach. A third scatterer, was added randomly to some of the cases with its center at [8.05, 8.05]. The size of the scatterers as well as the frequency of the excitation source were selected randomly for simulations at each of the source locations. The colormap represents the location errors in meters. The (0,0) point corresponds to the coordinate center (O) shown in Fig. 1. The x and y labels for each of the cells are the coordinates of the bottom-left corner of the grid cell.

The evaluation results showed the median and mean for the location error to be 258 m and 429 m, respectively. The obtained results in Fig. 8 reveal similar location accuracies to the ones achieved when using fixed parameters for the source and the scatterers. The results show that the proposed approach still yields reasonable performance even when the source and the medium are only partially known and modeled.

Furthermore, the model finds its way to the source by looking at the disturbances caused by reflections from the scatterers inside the images of the 2D electric field profile. Our further analysis showed that the presence of such asymmetries inside the region is a requisite for the EMTR/ML approach to determine the 2D location of the source. So far, the study in Section I.C has been done considering two scatterers inside the medium. Once reducing the number of scatterers to only one, in the general case there would be an ambiguity in determining the position of the source with the data from only one sensor. The reason is that, with only one symmetrical scatterer present inside the medium as shown in Fig. S6, the sensor would receive the same signal if the excitation source is put at point A or A’, where A is an arbitrary point in the computational domain and A’ is the mirror of A with respect to the symmetry line. In the case of having only one scatterer, the aforementioned ambiguity could be resolved by adding a magnetic field sensor at the place of the existing electric field sensor to determine the direction of arrival. Furthermore, the ambiguity would not exist if the scatterer itself were asymmetrical. As a result, the minimum number of scatterers is one. In case of symmetrical scatterers, the minimum number to avoid ambiguities increases to two.

### Case study 2: Application to locate lightning flashes

Several methods allow to estimate the location of lightning using single-station measurements. They typically use the Poynting vector to determine the direction to the lightning and characteristics of the received electromagnetic fields to estimate the distance to the lightning. A review of single-station techniques is given in Rafalsky *et al*.^37^.

Note that existing single-station techniques require at least two different sensors to operate and they suffer from poor accuracy. To obtain mean location accuracies of the order of a few hundred meters, an array of multiple stations is used^1–3,11,38^. In what follows, we illustrate how the proposed algorithm in Section I.C can be used to locate cloud-to-ground lightning flashes in the Säntis region with acceptable accuracy using only data from a single electric field sensor at a single station. What follows will briefly describe the experimental electric field measurement system, the applied methodology, and the experimental validation results.

#### Electric field measurement system at Herisau

A study of the incidence of lightning to different towers in Switzerland showed the Säntis Tower is struck by lightning some 100 times a year^39,40^, making it far and away the most struck structure in the country. This telecommunications tower has a height of 123.5-m and it was erected in 1997 on the mountain from which it takes its name, the 2502-m Mount Säntis in the in northeastern part of Switzerland.

Sensors to measure the current and the current derivative from direct lightning strikes to the tower were installed at two heights and have been in operation since 2010^39,41,42^.

Sensors to measure the vertical electric fields and the horizontal magnetic field from lightning flashes to the tower were set up at a distance of 14.7 km in 2014, on top of a 25-m tall building in Herisau^42^. The wideband electric and magnetic sensors, including fiber optics for the signal transmission, were manufactured by Thomson CSF (currently Thales). The operation frequency of the vertical electric field measurement subsystem goes from 1 kHz to 150 MHz. Since the return stroke field waveforms are bandlimited to a few MHz^43^, we used a digitization rate of 50 MS/s, which is sufficiently high to avoid aliasing. The magnetic field measurement system has the same upper cut-off frequency but its lower cutoff is at 2 kHz^42^.

#### Implementation of the combined EMTR and ML approach

The experimental validation of the proposed combinational approach was carried out using data from a vertical electric field sensor installed at Herisau, at 14.7 km from the Säntis tower.

**Figure 9:**
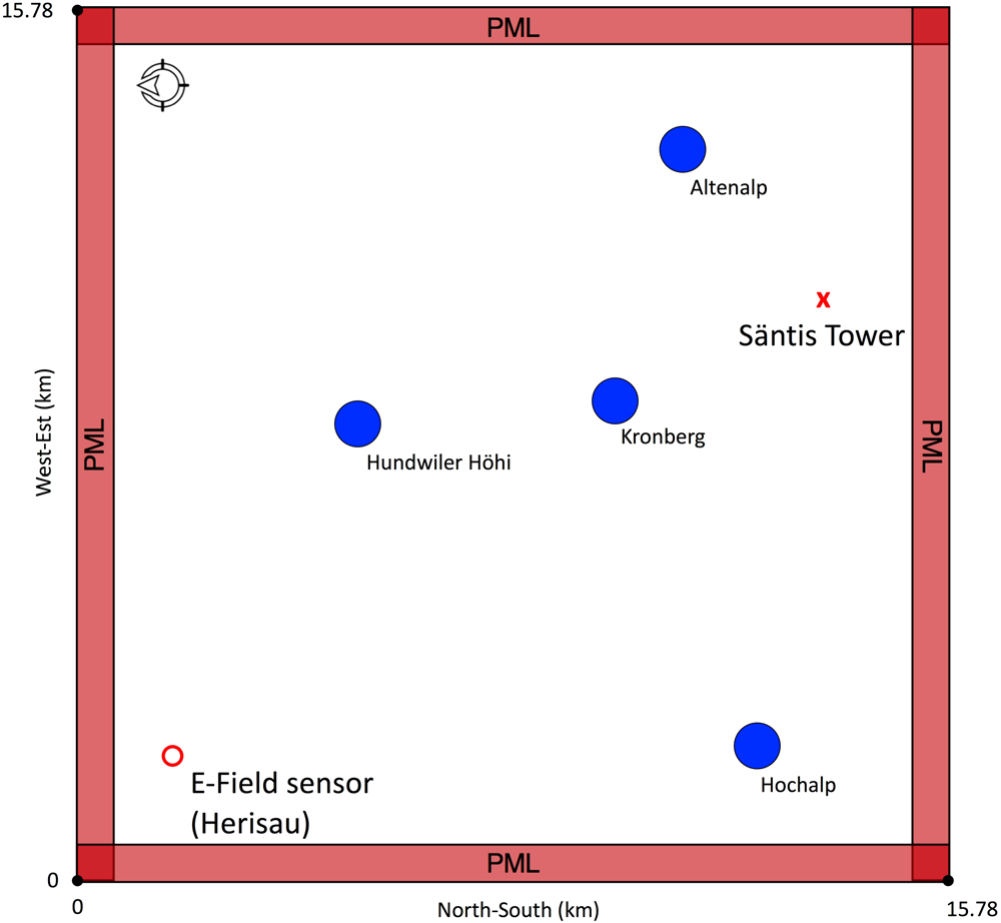
Geometry of the problem in the second case study, the blue/filled circles represent four actual tall mountains around the Säntis, modeled as cylinders. The red/unfilled circle shows the electric field sensor at Herisau. The red cross is the position of the Säntis Tower. The solution space spans 15.78 × 15.78 km^2^.

Consider a 15.78 × 15.78 km^2^ geographical grid including the Säntis Tower as shown in Fig. 9. We consider the electric field measurement system at Herisau to be the required sensor by the EMTR method. Four actual tall mountains around the Säntis were modeled as cylindrical scatterers. The scatterers had a radius ${\boldsymbol{r}}=240\,{\boldsymbol{m}}$, and electrical parameters ${{\boldsymbol{\varepsilon }}}_{{\boldsymbol{r}}}=10$, and ${\boldsymbol{\sigma }}=0.05\,{\boldsymbol{S}}/{\boldsymbol{m}}$. The geographical coordinates of the elements shown in Fig. 9 are given in Table 1.

**Table 1:** Coordinates of the elements shown in Fig. 9.

Item	Description	Latitude (°N)	Longitude (°E)
*Säntis Tower*	Lightning strike point	47.25	9.34
*Herisau*	Electric field sensor	47.39	9.27
*Altenalp*	Scatterer	47.27	9.38
*Hochalp*	Scatterer	47.28	9.25
*Hundwiler Höhi*	Scatterer	47.34	9.33
*Kronberg*	Scatterer	47.29	9.33

The European Cooperation for Lightning Detection (EUCLID)^44^ is a cooperation of several national lightning location networks aiming at providing lightning data all over Europe. The Säntis area is covered by six EUCLID sensors. In 2016, Azadifar *et al*.^45^ analyzed the performance of EUCLID for 269 upward lighting flashes striking the Säntis Tower from June 2010 to December 2013. The recorded flashes contain 2795 pulses (including return strokes or Initial Continuous Current pulses with a risetime lower than 8 µs and an amplitude greater than 2 kA). The results (see Fig. 11) showed the median, mean and maximum pulse location errors to be 186 m, 447 m, and above 5000 m respectively.

**Figure 10:**
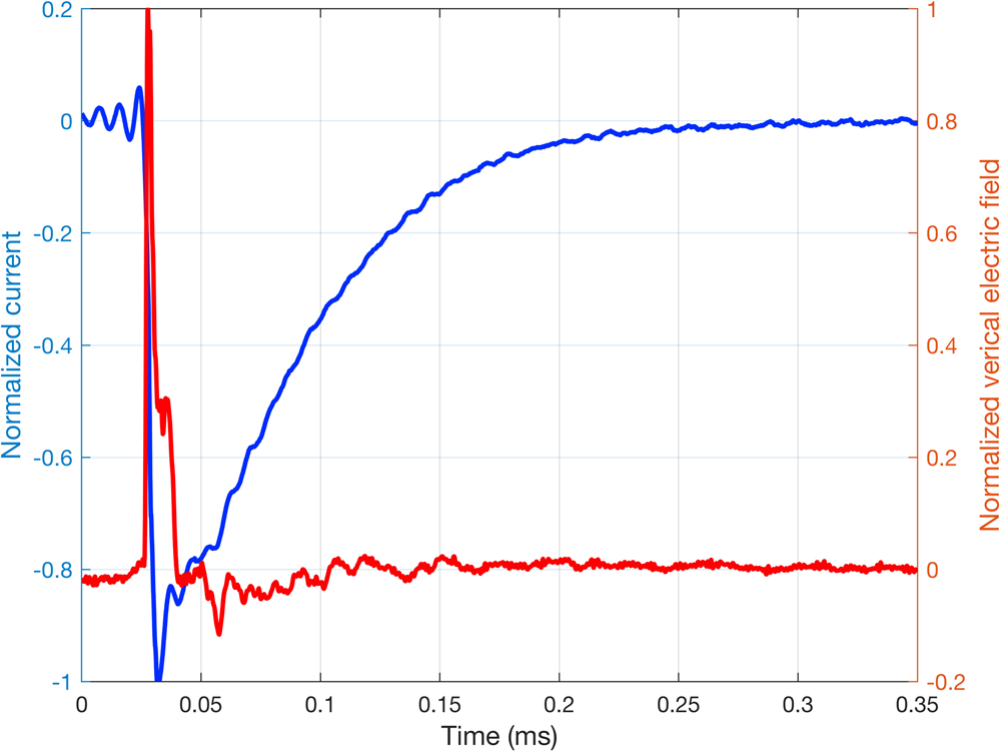
Simultaneous recordings of the vertical electric field at Herisau and the channel-base current associated with a return stroke of an upward lightning flash that occurred on on July 18^th^, 2017 at 18:31:35 (local time) at the Säntis Tower. The vertical electric field data were used for the experimental validation of the proposed approach.

In this study, the source positions used to build the database were taken from the EUCLID lightning location network in the region shown in Fig. 9 between March 2016 to July 2017. The database was built from 1296 geolocations that were randomly selected out of all of the geolocations in that region and time period. The lightning was modeled as a z-axis dipole current source excited by a Heidler current whose parameters are those used in Karami *et al*.^46^. The ML model (which consists of layers from VGG-19 with pretrained weights and the added regressors at the top) were then trained on the database. As the testing set, we used the experimental records of the electric field at Herisau for six return strokes (hereafter called RS1-RS6) corresponding to two upward negative lightning flashes occurred on October 21st, 2014 at 20:23:22 (local time) and July 18th, 2017 at 18:31:35 (local time). Figure 10 shows the electric field waveform associated with RS1. More information about the characteristics of the current waveforms of these return strokes is given in Table 2. In order to make the experimental data compatible with the input of the model, we carried out the following steps: (i) denoised the signals using MATLAB’s Wavelet Signal Denoiser^47^ and normalized the signal to its maximum value, (ii) time-reversed the normalized signal numerically and back-injected it into the medium using the 2D-FDTD method described in Section I.A.1 and the geometry shown in Fig. 9, and (iii) generated an RGB image out of the 2D data inside the detection region. Once the image was generated, the pretrained VGG-19 model was used to extract the feature vectors from it. This vector was then regarded as the testing sample and it was passed to the fitted model to evaluate its performance. In the second case study, the complications arising due to the sensor noise and weather conditions made the final regression problem have a higher complexity level. As a result, the accuracy of a neural network with a single point of non-linearity in the activation function deteriorates, making it unsuitable for solving the localization problem. Increasing the number of layers may allow incorporating more complicated models, but successful training of such a network requires much more training data than available. This led us to consider gradient boosting as an alternative, which makes it possible to estimate highly nonlinear functions with relatively fewer samples. An ensemble of 100 trees with depths capped to 3 to avoid overfitting achieved the best result for estimating the x and y coordinates in this problem.

**Figure 11:**
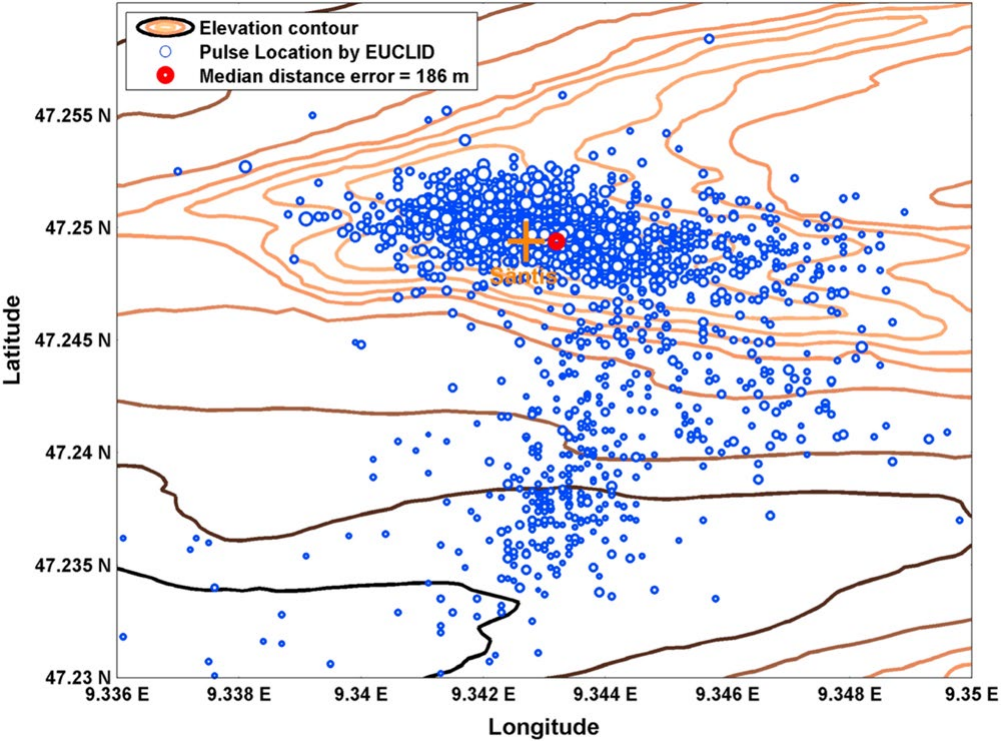
EUCLID pulse location errors for upward negative flashes striking the Säntis Tower recorded in the period of June 2010 until December 2013. The ground truth target for all pulses is the Säntis Tower and the estimated locations by EUCLID are presented as blue dots. The size of the dots is proportional to the pulse peak current amplitude. The length and width of the shown area are, respectively, 3.34 and 1.06 km. The figure is adopted from Azadifar *et al*.^45^.

**Table 2:** Characteristics of the current waveforms associated with the considered 6 return strokes (RS1 to RS6) and their corresponding location error using the EMTR/ML approach.

Return stroke	Peak amplitude current (kA)	Zero to peak rise time (µs)	Full width at half maximum (FWHM) (µs)	Location error (m)
RS1	12.4	8.2	52.2	365
RS2	4.5	5.5	50.8	347
RS3	3.4	5.8	48.3	330
RS4	2.7	5.6	44.9	325
RS5	8.1	5	114	39
RS6	13.2	1.3	29.1	113

#### Experimental validation results

**Figure 12:**
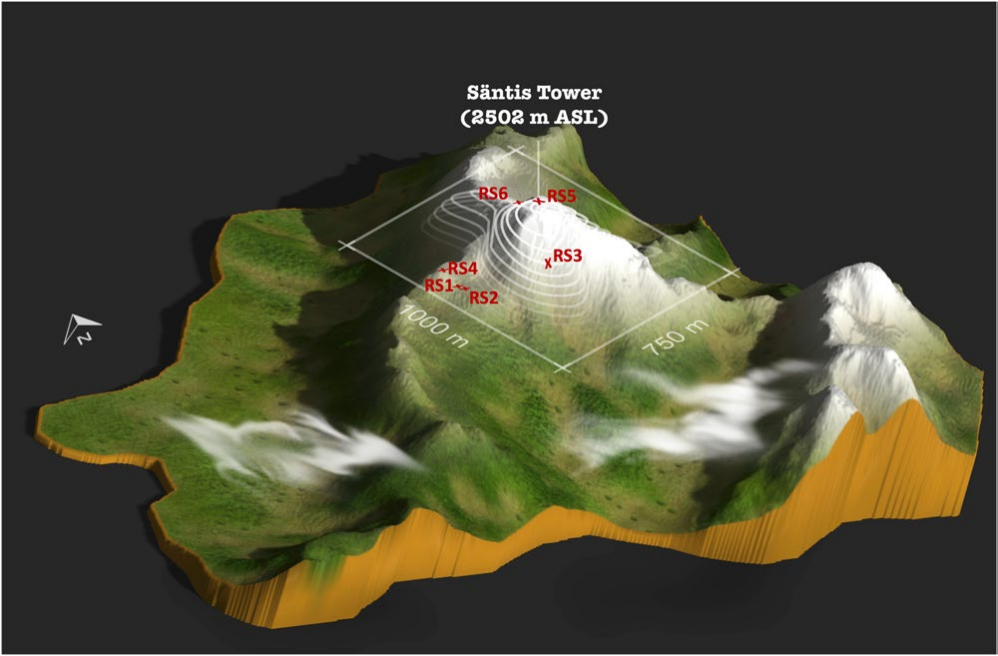
Experimental validation result for the proposed combinational approach: six return strokes (RS1-6) associated with two upward lightning flash that occurred at the Säntis Tower were the excitation source and the single-sensor recording of the associated electric fields 14.7 km away were the input data for the model. The estimated lightning strike point for each of the RSs is also shown as a red cross around the target (i.e., the Säntis Tower). The map is generated using 3D Map Generator–Atlas plugin (https://graphicriver.net/item/3d-map-generator-atlas-from-heightmap-to-real-3d- map/22277498) for Adobe Photoshop CC 2017.1.1 release.

The location errors for each of the tested RSs are reported in Table 2. The model’s prediction can also be seen visually in Fig. 12. As mentioned above, the task was to estimate the lightning strike point (i.e., the Säntis Tower) by looking at the single-sensor measurement of its associated electric field recorded 14.7 km away from the lightning channel. It can be seen that the model was able to locate the strike point with mean and median errors of 253 m and 328 m, respectively. The achieved result can be interpreted as a very good estimation skill for the model given the facts that (i) the detection area is mountainous, which highly affects the wave propagation inside the medium and (ii) only a simplified modeling of the geometry was used during the 2D-FDTD simulations.

A discussion is in order concerning the experimental validation and the conditions under which the combinational method presented here is applicable.

Although the performance of the combinational method in terms of location accuracy is excellent, both in the simulations and based on measured electric field data, further experimental confirmation is needed before any definitive conclusions can be drawn concerning the location accuracy of the method since lightning flashes at one source position were used for the experimental validation.

Finally, note also that, as mentioned in Section I.C, the fact that the terrain contains mountains or other scatterers is a requirement for the proposed combinational method to locate the discharges.

## Discussion

In this paper, machine learning and electromagnetic time were paired to introduce a new single-sensor electromagnetic source localization technique. In the proposed combinational model, on the one hand, EMTR is used as a preprocessing stage to convert a measured transient electric signal into more relevant input data (i.e., corresponding more to the target of determining the location of the source) for the ML model. On the other hand, machine learning is used to fit the back-propagation results to the target without any explicit underlying teleconnections available in the data. The method requires the presence of at least one scatterer as long as its shape is asymmetrical. In case of symmetrical scatterers, the minimum number to avoid ambiguities increases to two.

A 2D-FDTD method was used to calculate the electric field distribution on the detection region and generate the 2D profiles of vertical electric field as RGB images. Next, employing transfer learning, a pretrained VGG-19 Convolutional Neural Network (CNN) was used as the feature extractor tool and it was fed by the aforementioned simulation-generated images. Finally, two regressors were trained on the extracted features to do the final estimation of the source position inside the solution panel.

The method was first illustrated using a numerical example to localize a Gaussian RF source with 0.1–10 MHz bandwidth. A sensitivity analysis was presented using alternative parameters, such as the size and number of scatterers and the frequency of the excitation source.

The proposed method was then applied to the localization of lightning discharges in the Säntis region in Northeastern Switzerland. The model was trained based on the simulation results and tested using experimental observations of lightning flashes in the Säntis region. The experimental validation results show a high estimation accuracy for the combinational approach in finding the 2D geolocation of the lightning strike point at the Säntis Tower using only one sensor. The comparison to the results from EUCLID (given in Section II.B) reveals that the proposed approach can yield similar location accuracy with a significantly smaller number of sensors.

An extensive amount of work is in progress by the authors to further validate the proposed approach using more experimental cases and to consider 3D modeling to better simulate the medium and the excitation source details.

## Supplementary information

Supplementary Information

## Data Availability

The data that support the findings of this study are available upon reasonable request from the corresponding author.
