# Supplementary material for: Single-Sensor Source Localization Using Electromagnetic Time Reversal and Deep Transfer Learning: Application to Lightning
Source: Sci Rep. 2019 Nov 22;9:17372. doi: 10.1038/s41598-019-53934-4 (PMC6874538; doi:10.1038/s41598-019-53934-4)
Supplement: Supplementary file 1 — Supplementary Information [file 41598_2019_53934_MOESM1_ESM.pdf]

Supplementary Information

Single-Sensor Source Localization Using Electromagnetic Time Reversal and Deep Transfer Learning: Application to Lightning

Amirhossein Mostajabi[1], Hamidreza Karami[1], Mohammad Azadifar[2], Alireza Ghasemi[3], Marcos Rubinstein[2], Farhad Rachidi[1]

[1]Electromagnetic Compatibility Laboratory, Swiss Federal Institute of Technology (EPFL), Lausanne, Switzerland

[2]Institute for Information and Communication Technologies, University of Applied Sciences of Western Switzerland (HES-SO), Yverdon-les-Bains, Switzerland

[3]ELCA Informatik AG, Zürich, Switzerland

Corresponding author:

Farhad Rachidi

ELL 138, ELL building, Station 11
1015 Lausanne, Vaud, Switzerland
Tel. +41 21 693 2620
Fax. +41 21 693 2661
farhad.rachidi@epfl.ch

Contents of this file

The supporting information contains six supplementary figures.

Figures

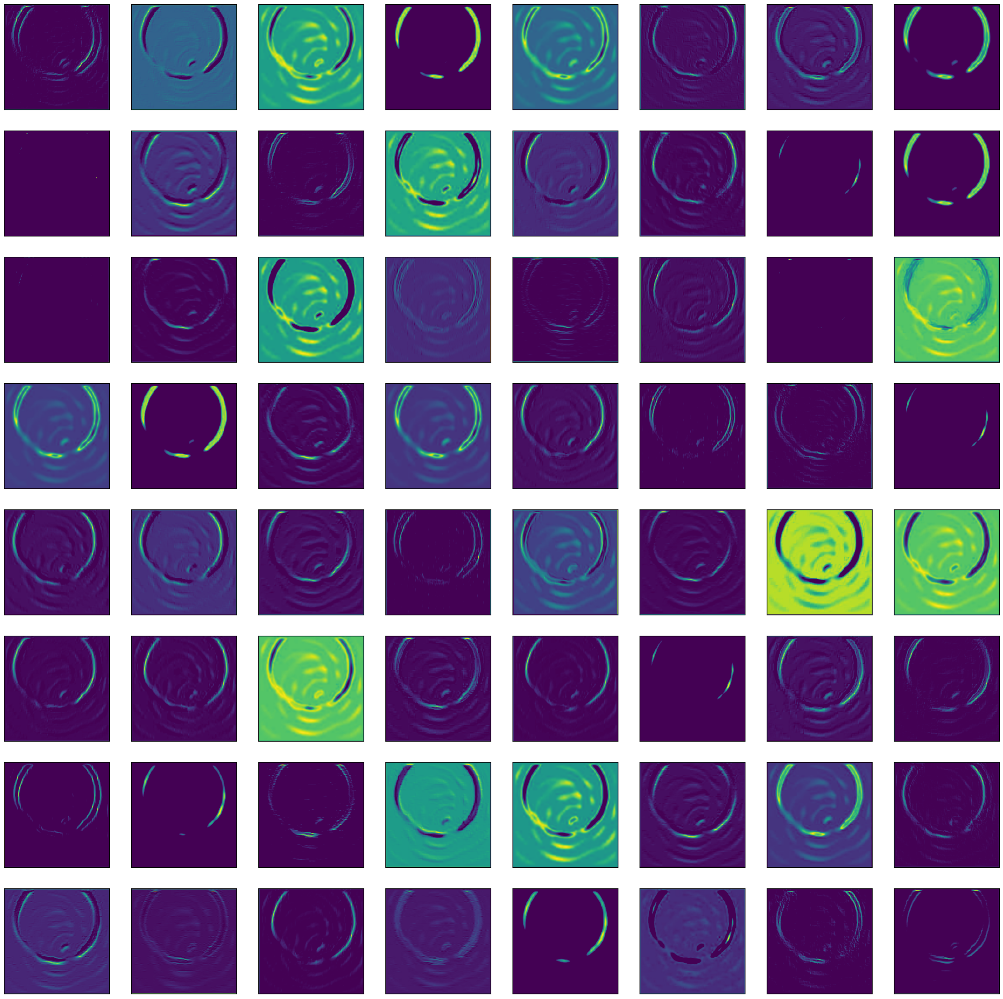

Fig. S1. Visualization of the first 64 feature maps of the 2nd layer in the VGG-19 model. The input image is the one presented in Fig. 5.

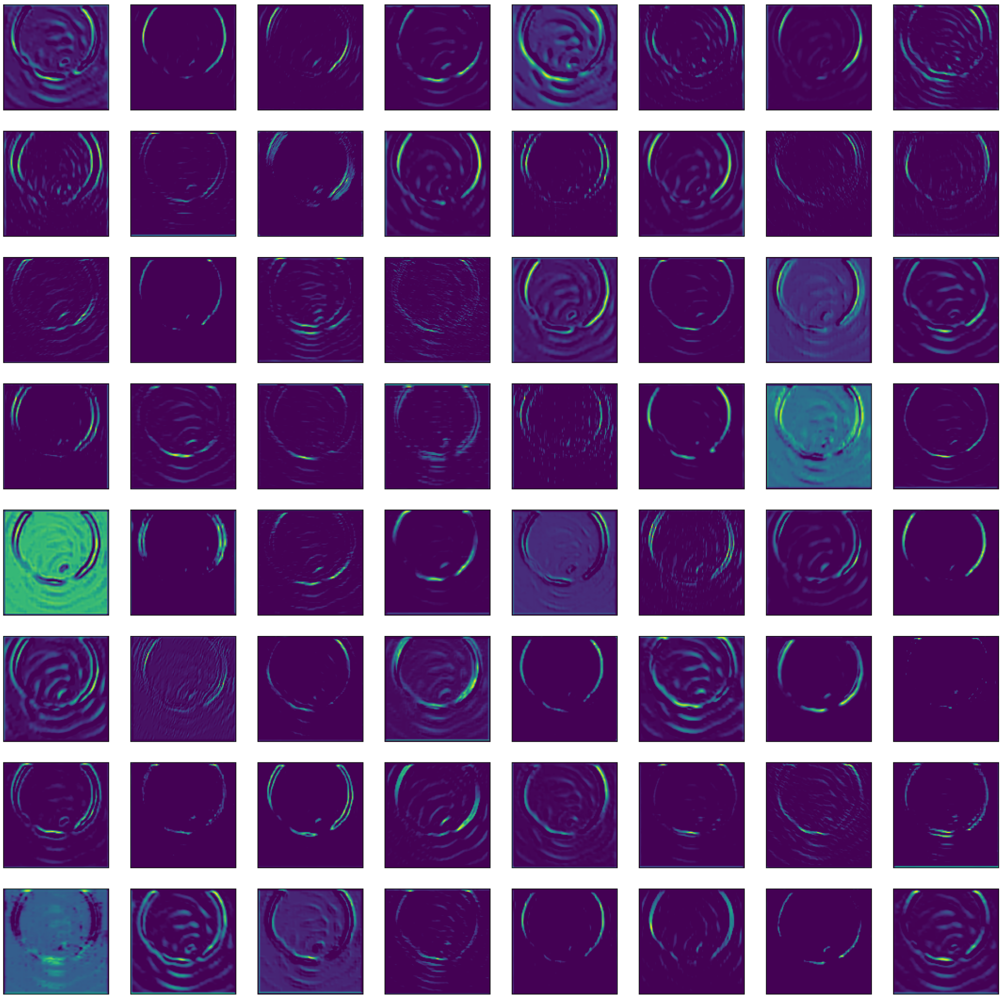

Fig. S2. Visualization of the first 64 feature maps of the 5th layer in the VGG-19 model. The input image is the one presented in Fig. 5.

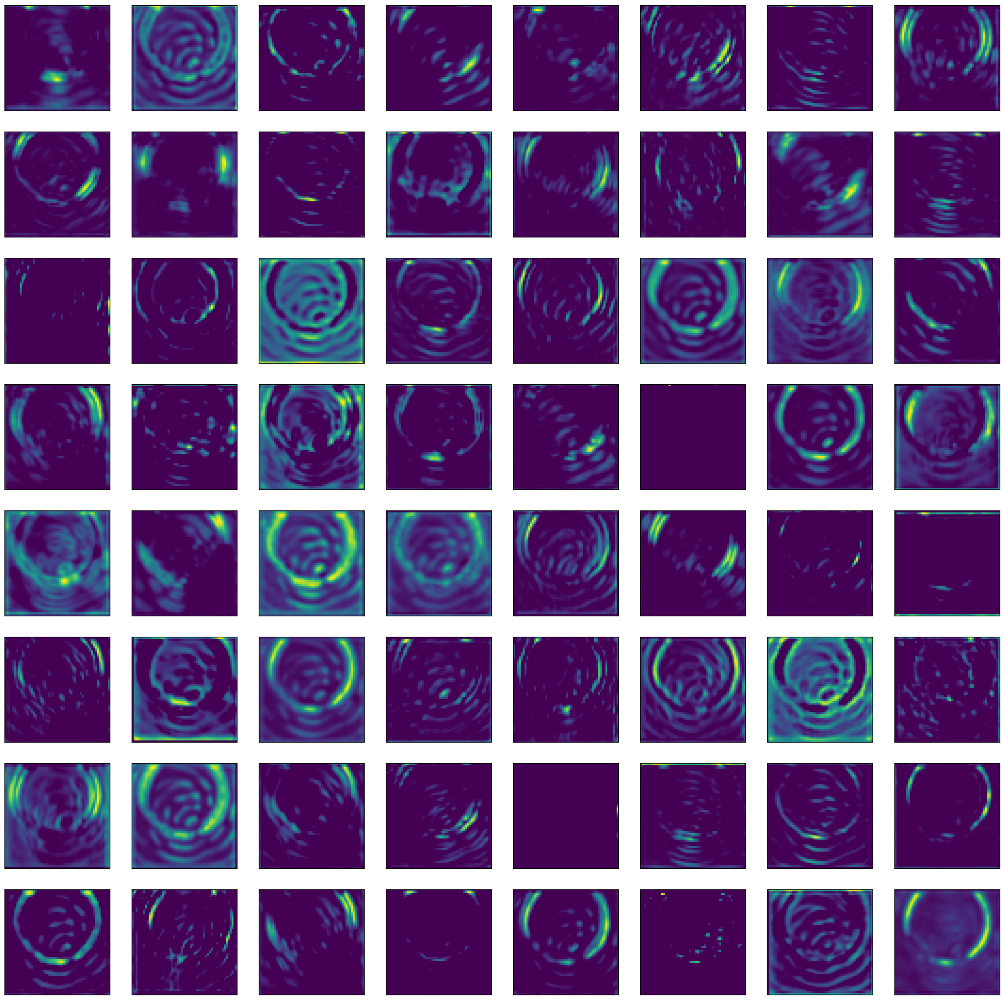

Fig. S3. Visualization of the first 64 feature maps of the 10th layer in the VGG-19 model. The input image is the one presented in Fig. 5.

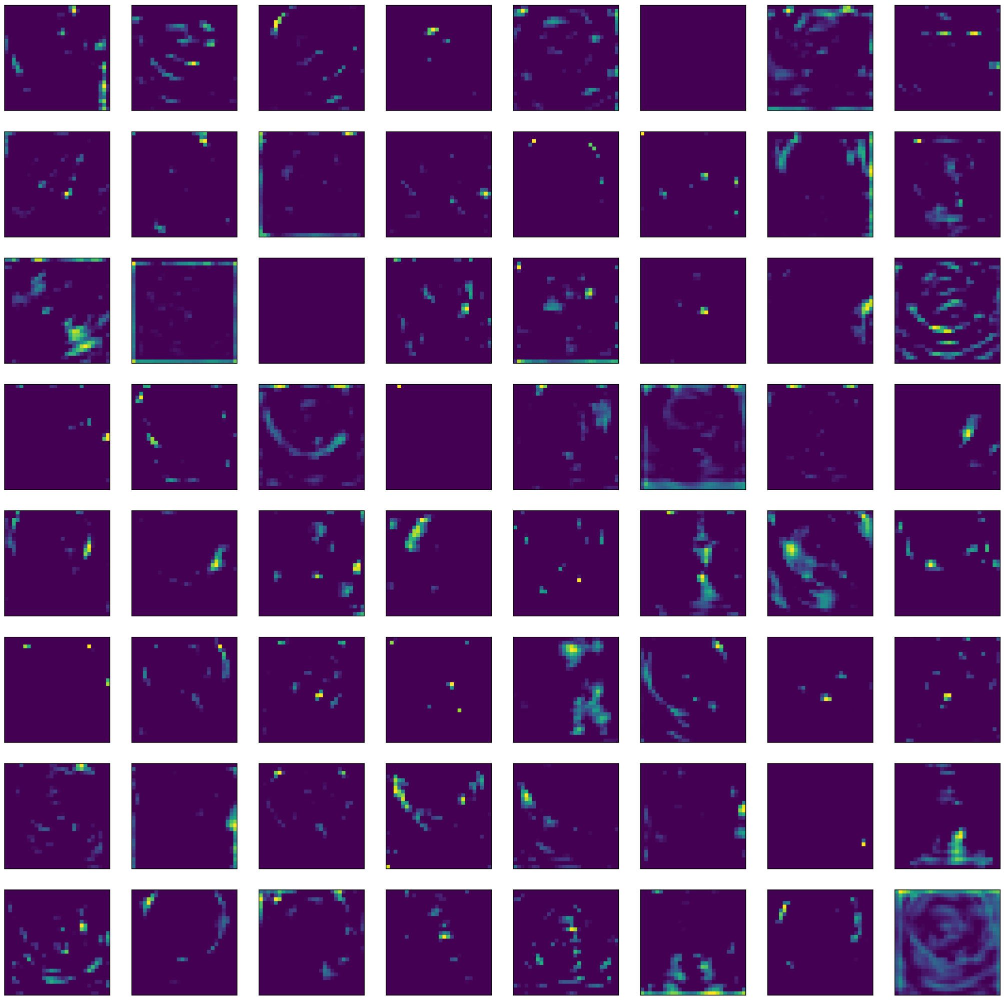

Fig. S4. Visualization of the first 64 feature maps of the 15th layer in the VGG-19 model. The input image is the one presented in Fig. 5.

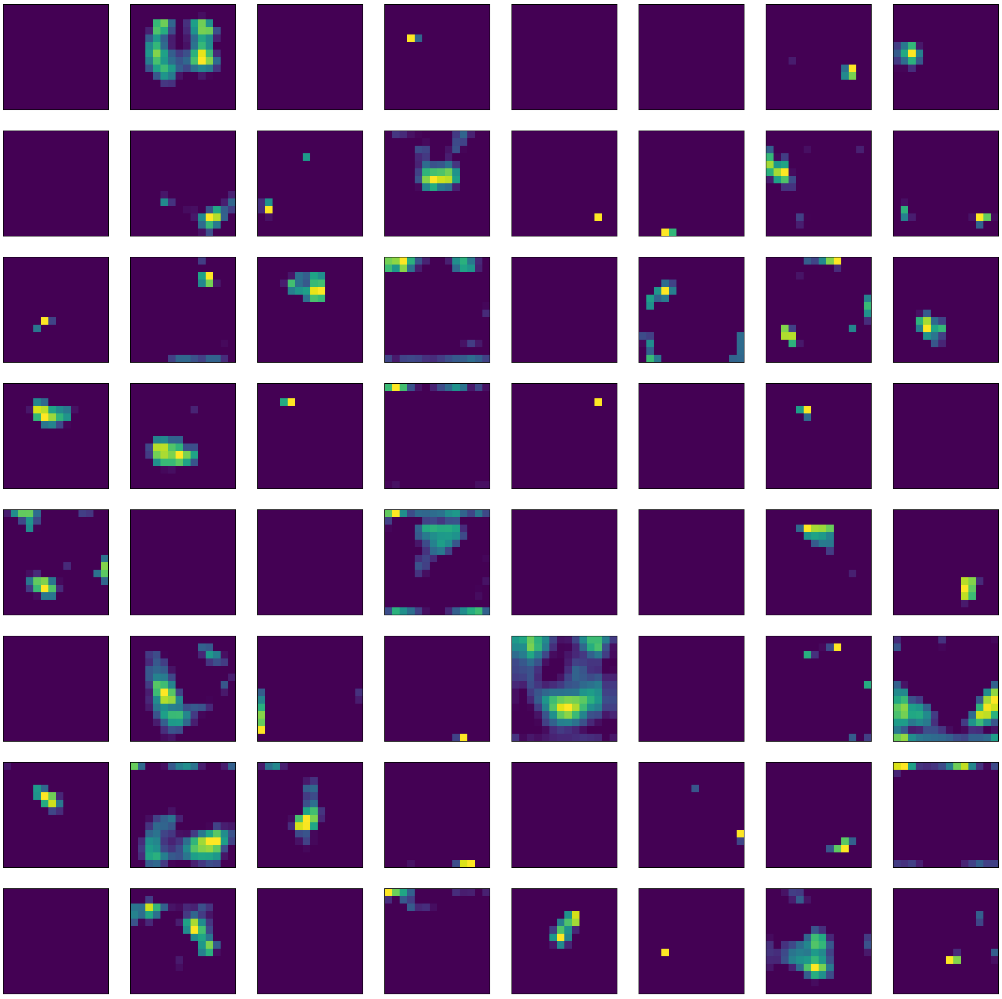

Fig. S5. Visualization of the first 64 feature maps of the 20th layer in the VGG-19 model. The input image is the one presented in Fig. 5.

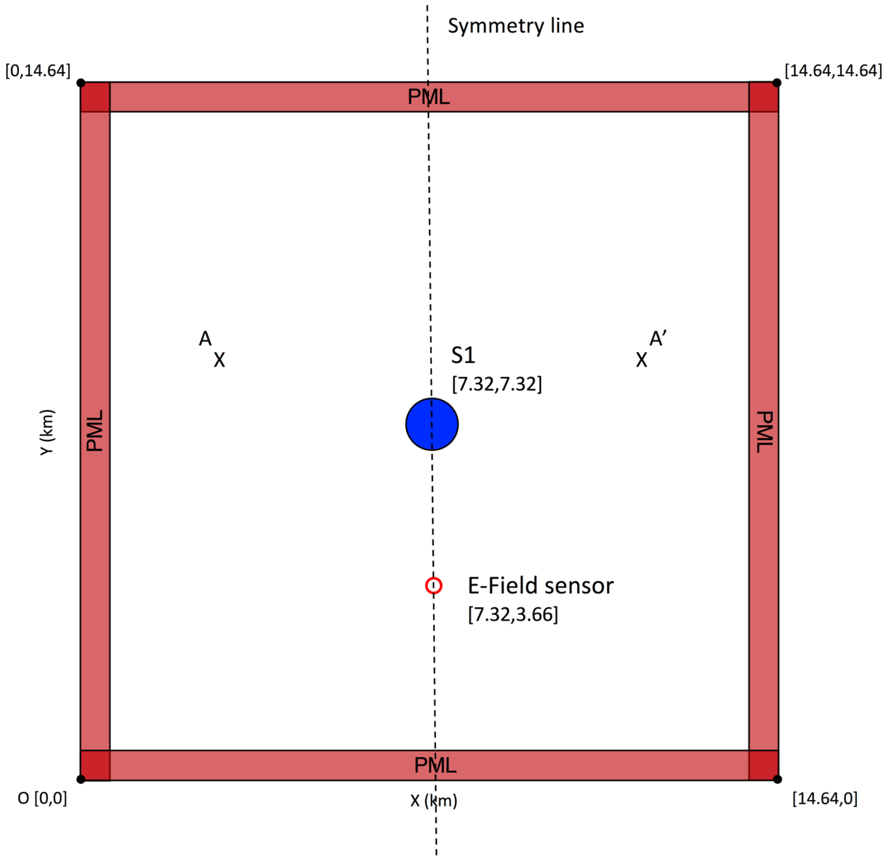

Fig. S6. Ambiguity of the solution in case of one symmetrical scatterer. A is an arbitrary point inside the medium and A' is the mirror of A with respect to the symmetry line.
